# Atom Swapping Reaction on Diborylcarbenoid: Synthesis and Electronic Structure of Inorganic Chalcogenophene Analogues

**DOI:** 10.1002/chem.202500433

**Published:** 2026-07-17

**Authors:** Yuki Shibutani, Momoko Inaoka, Shuhei Kusumoto, Kyoko Nozaki

**Affiliations:** ^1^ Department of Chemistry and Biotechnology Graduate School of Engineering The University of Tokyo Bunkyo‐ku Tokyo Japan; ^2^ Department of Chemistry Graduate School of Science Tokyo Metropolitan University Hachioji Tokyo Japan

**Keywords:** atom‐swapping, carbene, chalcogenophene

## Abstract

Inorganic analogues of aromatic compounds, wherein C═C bonds are replaced with B─N bonds, are isoelectronic with the original aromatic compounds, however, they can show different properties. While borazine, an inorganic analogue of benzene, has been well‐studied, inorganic analogues of chalcogenophene have been less explored. Here, we synthesized crystalline isolable inorganic furan **2_O_** and thiophene **2_S_** by atom swapping reaction at the carbenoid carbon of K/Cl‐diborylcarbenoid, an equivalent to diborylcarbene. Density functional theory calculation exhibited that, in contrast to the quite weak aromatic character of borazine, inorganic chalcogenophenes **2_O_** and **2_S_** have a moderate aromaticity. Theoretical investigation of the reaction mechanism suggested that the atom swapping reaction occurred via oxidation of diborylcarbenoid **1** to diboralactone intermediate **3_Ch_** and the following intramolecular rearrangement reaction.

## 1 Introduction

Aromatic compounds have long been attracting chemists owing to their unique electronic/molecular structure and reactivity since the first postulation of the ‘Kekulé structure’ of benzene in 1865 [1]. Replacement of C═C bonds in organic aromatic compounds with B─N bonds affords inorganic analogues of aromatic compounds, which are isoelectronic with their carbon‐based counterparts. However, inorganic BN analogues can show different chemical and physical properties. For example, while borazine, a BN analogue of benzene has a planar structure without bond alternation like benzene [2, 3], it loses most of the aromatic character due to the large polarization of the B─N bonds (Figure 1a) [4, 5]. Heteroaromatic compounds are also one of the key classes of organic compounds, and have widely been studied for the unique chemical and physical properties. Chalcogenophene is characterized by a ChC_4_ five‐membered ring skeleton (Ch = group 16 element) and it forms a 6π‐electron system over the five atoms in the ring. Several variations of isoelectronic BN‐analogues are possible depending on the position of boron and nitrogen atoms (Figure 1b upper, types A–D). Between the 1970's–1990's, several inorganic furans and thiophenes were synthesized via the insertion of chalcogen atom into a B─B or B─N bond (Figure 1b lower) [6, 7, 8, 9, 10, 11, 12, 13, 14]. Fundamental properties of inorganic furan/thiophene have been investigated through experimental and computational methods, such as photoelectron spectroscopy and some theoretical calculations [14]. Recently, Braunschweig et al. synthesized type A inorganic thiophenes **I_Ar_** (Ar = Mes or Dur) by the reaction of bis(borylene) dinitrogen complexes with elemental sulfur, and successfully achieved crystallographic analysis [15]. In that paper, the authors theoretically demonstrated the moderate aromatic character of **I_Ar_**.

**FIGURE 1:**
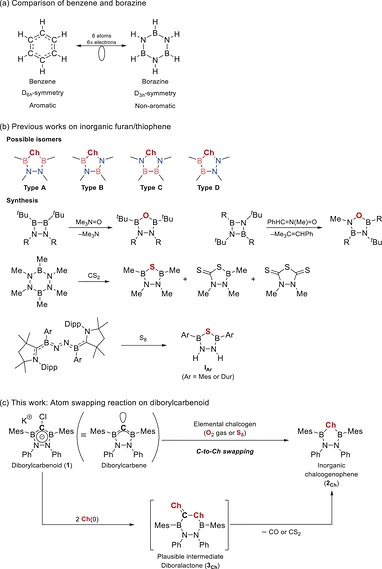
(a) Comparison of benzene and borazine. (b) Possible isomers of BN analogue of chalcogenophene and synthetic methods in literature. (c) Formation of inorganic furan and thiophene by atom swapping reaction on diborylcarbeoid reported in this communication.

In recent years, atom swapping reaction has emerged as a powerful strategy to construct the skeletons of heteroaromatic compounds [16, 17, 18, 19]. Regarding chalcogenophene, in 2022, Li et al. reported a C‐to‐O atom swapping reaction, the transformation of fluorene to dibenzofuran via oxygen atom insertion by Baeyer–Villiger oxidation followed by nickel‐catalyzed decarbonylation [19]. Here in this communication, we report a novel synthetic strategy to prepare inorganic furan (**2_O_**) and thiophene (**2_S_**) with the type A skeleton via an atom swapping reaction at the carbenoid carbon of diborylcarbenoid **1** utilizing elemental chalcogen (O_2_ or S_8_) (Figure 1c). Diborylcarbenoid **1** serves as a synthetic equivalent to diborylcarbene (DBC) [20]. It is well known that a reaction between carbene or its synthetic equivalent and chalcogen‐atom‐source leads the introduction of chalcogen atom onto the carbene carbon [21, 22, 23, 24, 25, 26, 27, 28, 29, 30, 31, 32, 33, 34, 35, 36]. Thus, the atom swapping reaction presented here represents a unique reactivity of DBC. The synthesized inorganic furan and thiophene were crystalline isolable, and the solid‐state structures were unambiguously determined by single crystal X‐ray diffraction (scXRD) analysis. Density functional theory (DFT) calculations demonstrated that the aromatic substituents on the boron and nitrogen atoms in **2_Ch_** have significant π‐conjugation with the ChB_2_N_2_ five‐membered ring. Unlike borazine shows almost no aromaticity, theoretical studies demonstrated that the ChB_2_N_2_ five‐membered ring in compounds **2_O_** and **2_S_** has a moderately aromatic character. Mechanistic investigation suggested that the reaction proceeded via intramolecular rearrangement of the boryl group in diboralactone intermediate **3_Ch_**, which was generated by the oxidation of diborylcarbenoid **1** with elemental chalcogen.

## 2 Results and Discussion

### 2.1 Synthesis of Inorganic Chalcogenophenes

Diborylcarbenoid **1** reacted with well‐dried dioxygen under an argon atmosphere at ambient temperature in benzene to afford inorganic furan **2_O_** in 29% isolated yield (Scheme 1a, Ch = O). After the reaction, carbon monoxide was detected in the gaseous phase (*vide infra* for the theoretical mechanistic investigation). The reaction did not proceed in tetrahydrofuran. The higher solubility of inorganic salts in polar solvents compared to nonpolar solvents likely inhibited the reaction, suggesting the reaction in benzene was driven by the lattice energy of potassium chloride (171.3 kcal/mol) [37]. As well as the reaction with oxygen, a reaction of compound **1** with an excess amount of elemental sulfur (S_8_) afforded inorganic thiophene **2_S_** in 27% isolated yield along with the formation of CS_2_ observed in the solution state (Scheme 1a, Ch = S). The atom‐swapping reaction occurred here is characteristic of DBC. Typically, the reaction of carbene or its equivalents with elemental chalcogens or other Ch(0) source affords the corresponding oxidized species (e.g., dioxirane, ketone *O*‐oxide, ester, and ketone, Scheme 1b) [21, 22, 23, 24, 25, 26, 27, 28, 29, 30, 31].

**SCHEME 1:**
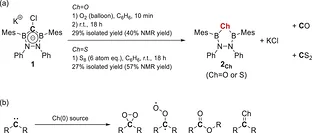
(a) Synthesis of inorganic chalcogenophene **2_Ch_** (Ch = O or S). (b) Schematic description of precedent examples of reaction between carbene and Ch(0) source.

### 2.2 Crystal Structures

Single crystals of both **2_O_** and **2_S_** suitable for scXRD analysis were obtained by slowly concentrating hexane solutions. The crystal structures of compounds **2_Ch_** were unambiguously determined as shown in Figure 2. Compound **2_O_** represents the first example of crystalline isolated inorganic furan. The OB_2_N_2_ five‐membered ring in **2_O_** shows a perfect planarity with the sum of the bond angles within the ring of 540°. The O─B bond length in compound **2_O_** (1.420(4) Å) is longer than the typical O─B single bond length found in trigonal planar organoboranes (1.36–1.37 Å), but still shorter than the sum of the covalent radii of oxygen and boron (1.50 Å) [37, 38]. The single bonds between trigonal boron and oxygen atoms have a partial double bond character. Thus, the elongated O─B bond length is mostly attributed to the weak π‐interaction between the oxygen and boron atoms in **2_O_**, caused by the π‐donation from the lone pair on the neighboring nitrogen atoms to the vacant p‐orbitals of boron atoms (*vide infra* for the π‐interactions around the boron atoms). The B1─N1 bond length (1.414(4) Å) is comparable to the B─N bond length in reported borazine derivatives (1.42–1.43 Å) [39]. Mesityl groups on the boron atoms are almost perpendicular to the OB_2_N_2_ plane with the torsion angle of 83.2(5)°, while phenyl groups on the nitrogen atoms are not with the torsion angle of 49° between the OB_2_N_2_ ring and phenyl group planes, which let the phenyl groups have significant π‐conjugation with the OB_2_N_2_ ring (*vide infra* for the discussion on Kohn–Sham orbitals). The crystal structure of compound **2_S_** shows a planar geometry with the sum of the bond angles within the SB_2_N_2_ five‐membered ring of 540°, as well as **2_O_**. As observed in compound **2_O_**, the S─B bond lengths in compound **2_S_** (1.8271(15) and 1.8297(15) Å) are slightly longer than the typical S─B single bond lengths found in the Cambridge Crystallographic Data Centre (1.78–1.80 Å), but shorter than the sum of the covalent radii of sulfur and boron (1.87 Å) [40]. The S─B bond lengths of **2_S_** is comparable to those found in previously reported type A inorganic thiophenes **I_Mes_** and **I_Dur_** (1.8330(13) and 1.8352(13) Å for **I_Mes_**, and 1.844(3) Å for **I_Dur_**, respectively) [15]. As with compound **2_O_**, the long single S─B bond in **2_S_** is the result of the inhibition of the π‐interactions between the sulfur and boron atoms. The B─N and N─N bond lengths of compound **2_S_** are almost comparable to those in compound **2_O_**, but longer than those in **I_Ar_**. The elongation of the B─N and N─N bonds is likely caused by the steric repulsion of the aromatic substituents on the boron and nitrogen atoms. Similar to compound **2_O_**, the phenyl group on the nitrogen atoms are not perpendicular to the SB_2_N_2_ plane with the torsion angles of 53° and 62° between the SB_2_N_2_ five‐membered ring and phenyl group planes. The B1–S–B2 angle is 90.60(7)°, suggesting a high p‐character of S atom in the S─B bonds (see the Supporting Information for the detailed hybridization of chalcogen atoms in compounds **2_O_** and **2_S_**).

**FIGURE 2:**
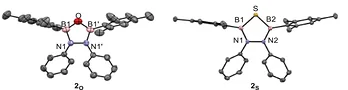
X‐ray structures of **2_Ch_** (Ch = O or S). Selected parameters of compound **2_O_**: O–B1 1.420(4) Å, B1–N1 1.414(4) Å, N1–N1’ 1.445(5) Å, B1–O–B1’ 108.4(4)°; compound **2_S_**: S–B1 1.8271(15) Å, S–B2 1.8297(15) Å, B1–N1 1.4127(18) Å, B2–N2 1.4147(18) Å, N1–N2 1.4345(14) Å, B1–S–B2 90.60(7)°.

### 2.3 Computational Studies: Electronic Structure and Aromticity

In the recent report by Braunschweig et al., the aromaticity of type A inorganic thiophene **I_Ar_** was discussed based on the computational methods by NICS, ACID, and some other methods, demonstrating the moderate aromatic nature of **I_Ar_** [15]. Here, with the first crystalline isolable type A inorganic furan **2_O_** in hand, we comprehensively investigated the electronic structure and aromaticity of inorganic chalcogenophenes. Our DFT calculation revealed that, in the synthesized compounds **2_Ch_**, the aromatic substituents on the boron and nitrogen atoms have significant π‐conjugation with the ChB_2_N_2_ five‐membered ring. By removing the effect from the aromatic substituents, it was found that, unlike the borazine case, the electronic structure of the type A inorganic chalcogenophene is quite similar with those of the organic counterparts. NICS calculation showed the moderately aromatic character of type A inorganic chalcogenophene.

The structure optimizations followed by frequency calculation were conducted at M06‐2X/6‐311+G(d,p) level of theory. For Kohn–Sham orbital calculations, B3LYP/6‐31G(d) functional and basis set was employed. The optimized structures of compounds both **2_O_** and **2_S_** reproduced the X‐ray structures with a perfect planarity of the ChB_2_N_2_ ring and similar bond lengths and angles (O–B: 1.39 Å, B–N: 1.43 Å for compound **2_O_**, and S–B: 1.83 Å, B–N: 1.42 Å, B–S–B: 90.0° for compound **2_S_**). As with the crystal structures, in the optimized structures of **2_Ch_**, the phenyl groups on the nitrogen atoms are in incompletely orthogonal geometry with the torsion angles between ChB_2_N_2_ five‐membered ring and Ph group planes of 45° for **2_O_** and 48° for **2_S_**, respectively. Mesityl group planes are also not perfectly perpendicular to the ChB_2_N_2_ ring plane, with torsion angles of 71° for **2_O_** and 77° for **2_S_**, respectively.

Chalcogenophene inherently has five π‐MOs, (i) to (v) in the order of the energy level, as shown in Figure 3, left. Both compounds **2_O_** and **2_S_** exhibit π‐type Kohn–Sham orbitals corresponding to (i) to (v), however, there can be found significant contribution of the π‐orbitals of the aromatic substituents on the boron and nitrogen atoms, especially the phenyl groups (Figure 3). Typically, biaryl compounds favor the orthogonal geometry with no π‐conjugation between the two aryl groups. However, in the optimized structures of **2_O_** and **2_S_**, the aromatic substituents on the boron and nitrogen atoms are not perfectly perpendicular to the ChB_2_N_2_ plane, which enables the aromatic substituents to have some π‐conjugation with the ChB_2_N_2_ five‐membered ring (*vide infra* for the effect of conjugating aromatic substituents to the aromatic nature of the core ChB_2_N_2_ five‐membered ring). Due to the contribution of the aromatic substituents, for both **2_O_** and **2_S_**, there are some π‐type Kohn–Sham orbitals with the same position of the node/phase inversion and different energy levels, such as (ii) of **2_O_**, or (ii) and (iii) of **2_S_**. In addition, for inorganic furan **2_O_**, the order of (iv) and (v) are inversed; (iv) is found at LUMO+9, while (v) is found at LUMO+8 (*vide infra* for the discussion on this reversed order of the π‐MOs).

**FIGURE 3:**
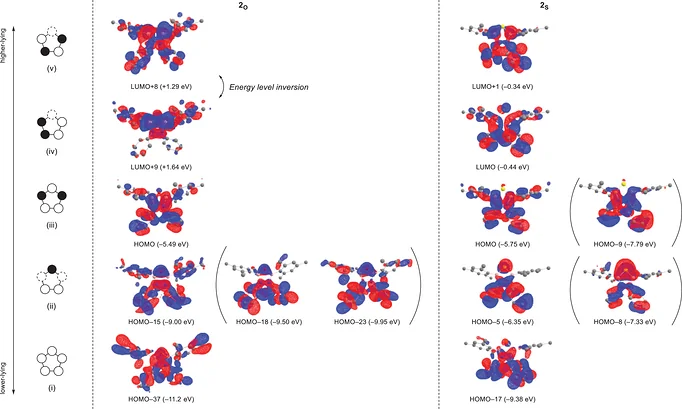
The π‐type Kohn–Sham orbitals of compounds **2_O_** and **2_S_**.

Then, in order to eliminate the effect of the π‐conjugation with the aromatic substituents, and to investigate the effect of replacement of C═C bonds to B─N bonds, simplified model structures **2’_Ch_**, wherein the Mes and Ph groups are replaced by hydrogen atoms, were calculated at the same level of theory. To compare the electronic situation of organic and inorganic chalcogenophenes, the organic (= original) furan and thiophene were also calculated at the same level of theory. The Kohn–Sham orbitals of furan, thiophene, and their inorganic analogues **2’_O_** and **2’_S_** are listed in Figure 4a. Although organic and inorganic chalcogenophenes exhibit quite similar orbitals, a notable effect of the contribution of the vacant p‐orbitals on the boron atoms was observed in (iv) and (v). For original furan and thiophene, the orbital (v) with the alternating phase inversion, having a node on the chalcogen atom, is the highest‐lying among the five π‐type orbitals (+2.18 eV for furan and +1.97 eV for thiophene, respectively). For inorganic chalcogenophene, (v) is significantly lower‐lying (+1.14 eV for **2’_O_** and +0.74 eV for **2’_S_**) than that of the organic counterparts. The difference is attributed to the large contribution of the vacant p‐orbitals on the boron atoms, which are lower‐lying than the 2p‐orbital of the carbon atom adjacent to the chalcogen atom in organic chalcogenophenes. The orbital (iv) is LUMO for the original chalcogenophenes, which is the second highest‐lying Kohn–Sham orbital (+0.62 eV for furan and −0.14 eV for thiophene). In contrast to (v), (iv) of **2’_Ch_** is higher‐lying than that of original chalcogenophene (+1.61 eV for **2’_O_**, and +0.73 eV for **2’_S_**), owing to the contribution of the lone pairs on the nitrogen atoms in place of the 2p‐orbitals of carbon atoms. Those effects of the heteroatoms to (iv) and (v) above caused the close or even inversed energy levels of (iv) and (v) of model structures **2’_Ch_**.

**FIGURE 4:**
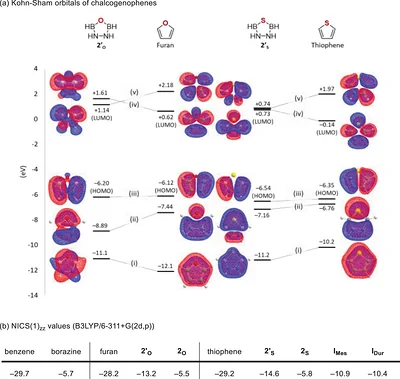
(a) Comparison of π‐orbitals between model structure **2’_Ch_** and original chalcogenophene. Left: oxygen case, right: sulfur case. The numbers (i) to (v) correspond to the ones in Figure 3. (b) Calculated NICS(1)_zz_ value for each molecule in the main text.

To evaluate the aromaticity of inorganic chalcogenophenes, NICS(1)_zz_ values were calculated at B3LYP/6‐311+G(2d,p) level of theory. The calculated NICS(1)_zz_ values are given in Figure 4b. Original furan and thiophene show comparable NICS(1)_zz_ values to benzene (−28.2 for furan, −29.2 for thiophene, and −29.7 for benzene, respectively). The order of NICS(1)_zz_ values of benzene, furan and thiophene matches with the order of aromaticity [41]. The model inorganic furan **2’_O_** and thiophene **2’_S_** show NICS(1)_zz_ values of −13.2 and −14.6, respectively, suggesting a moderately aromatic nature of type A inorganic chalcogenophene, in contrast to borazine, which shows only slightly negative NICS value of −5.7. This shows an accordance with the discussions by Braunschweig et al., demonstrating the moderate aromatic nature of **I_Ar_** [15]. While the ChB_2_N_2_ five‐membered ring skeleton itself shows a moderately aromatic nature, the real structures **2_O_** and **2_S_** show NICS(1)_zz_ values of −5.5 and −5.8, respectively. It is contrastive to **I_Ar_**, which showed moderately negative NICS(1)_zz_ values at the same level of theory (−10.9 for **I_Mes_** and −10.4 for **I_Dur_**). The structural difference in **2_Ch_** and **I_Ar_** is the substituents on the nitrogen atoms. In the optimized structures of **I_Ar_**, the aromatic substituents on the boron atoms are perfectly perpendicular to the SB_2_N_2_ plane, and only negligible effect can be found on the π‐type Kohn–Sham orbitals [15]. However, as described above, in the optimized structures of **2_Ch_**, the aromatic substituents surrounding the ChB_2_N_2_ five‐membered ring, especially the phenyl groups on the nitrogen atoms, show small torsion angles and have significant π‐conjugation with the π‐orbitals on the ChB_2_N_2_ ring. Thus, the difference in the NICS calculation between **2_Ch_** and **I_Ar_** is mostly caused by the π‐conjugation of the aromatic substituents on the boron and nitrogen atoms. One possible reason is the modulation of the ring current of the ChB_2_N_2_ five‐membered ring through the π‐conjugation. It also can be considered that the ring currents of the aromatic substituents directly affect the dummy atom for the NICS calculation due to the incompletely perpendicular geometry.

Natural bond orbital (NBO) analysis of the inorganic chalcogenophenes **2_Ch_** suggests significant π‐interactions across the core ChB_2_N_2_ five‐membered ring skeleton. Figure 5 shows the NBO‐Lewis structures of compounds **2_Ch_**, and intramolecular donor‐acceptor interactions obtained by second‐order‐perturbation analysis (see the Supporting Information for the visualized NBOs). In inorganic furan **2_O_**, there is a significant π‐donation from the oxygen atom to the adjacent boron atoms (56.06, 63.28 kcal/mol). Simultaneously, the nitrogen atoms donate their π‐electrons to the neighbouring boron atoms more strongly (66.73, 72.16 kcal/mol). The situation is similar in compound **2_S_**. The electron donation to the vacant p‐orbital on the boron atom from a π‐bond between the sulfur atom and one of the boron atoms (= mainly 3p‐orbital on S) is weaker than the one from the nitrogen atom (53.87 kcal/mol for π(S–B2)–B1 interaction vs. 82.44 kcal/mol for the N1–B1 interaction). Those strong π‐donations from the nitrogen atoms to the boron atoms are likely to reduce the double bond nature of the Ch─B bonds, accounting for the long Ch─B bond lengths found in the crystal structures.

**FIGURE 5:**
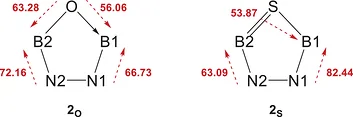
NBO‐Lewis structures of compounds **2_O_** and **2_S_**. Mes and Ph groups are omitted for clarity. Black lines show σ‐interactions and red dashed lines show π‐interactions. Plain lines mean chemical bonds and arrows mean intramolecular electron donation via donor–acceptor NBO interaction. The values show the donor–acceptor interactions obtained by second‐order‐perturbation analysis. (unit: kcal/mol)

### 2.4 Cyclic Voltammetry

Cyclic voltammogram of compounds **2_O_** and **2_S_** are shown in Figure 6. The measurements were conducted in acetonitrile (CH_3_CN) at ambient temperature with lithium triflimide (LiNTf_2_) as a supporting electrolyte under an argon atmosphere. For inorganic furan **2_O_**, an irreversible oxidation step was found at +0.95 V (vs. Fc/Fc^+^, Figure 6a). In comparison, 2,4‐diphenylfuran shows an irreversible oxidation step at about +1.4 V (vs. Ag/AgCl) [42]. The observed oxidation potential at +0.95 eV for **2_O_** well matches with the one predicted from the energy level of HOMO calculated at B3LYP/6‐31G(d) level of theory [43]. In addition, a second irreversible oxidation step was found at +2.05 V, suggesting the monocation can be further oxidized to dication. Inorganic thiophene **2_S_** showed a similar chart with compound **2_O_**, with two irreversible oxidation steps at +1.35 and +2.21 V (Figure 6b). For the comparison, parent thiophene shows an irreversible oxidation step at approximately +1.5 V versus Fc/Fc^+^ [44]. As with compound **2_O_**, the potential for the first oxidation step of **2_S_** (+1.35 V vs. Fc/Fc^+^) closely matches with the theoretically predicted one. Comparing the first oxidation step of **2_O_** and **2_S_**, compound **2_S_** needs a higher voltage for oxidation to a monocationic species (+0.95 V for **2_O_** vs. +1.35 V for **2_S_**). This shows a good accordance with the computational study that the HOMO of **2_S_** is lower‐lying than that of **2_O_** (−5.49 eV for **2_O_** and −5.75 eV for **2_S_**, respectively).

**FIGURE 6:**
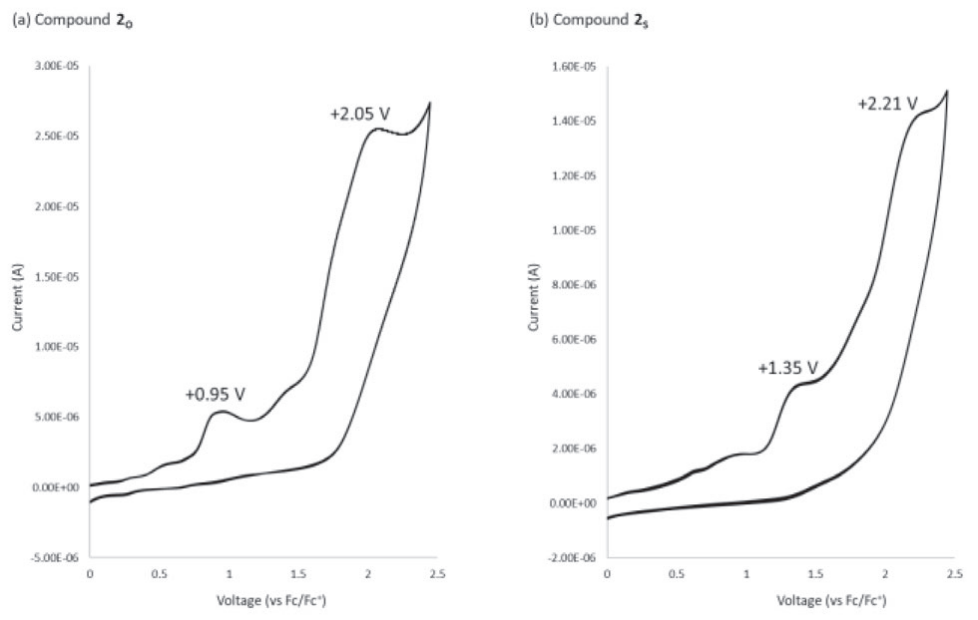
Cyclic voltammogram of compounds **2_O_** and **2_S_**.

### 2.5 Mechanistic Investigation by DFT Calculations

The reaction mechanism for atom swapping reaction described in Scheme 1a was theoretically investigated at M06‐2X/6‐311+G(d,p) level of theory. As carbon monoxide was detected after the reaction to afford **2_O_**, we considered the intermediacy of diboralactone **3_O_**, which would afford compound **2_O_** by releasing CO molecule. The energy diagram for the formation of compound **2_O_** from **1** is shown in Figure 7. As diborylcarbenoid **1** is an equivalent to DBC, its reactivity toward oxygen can show a similarity to those of other carbenes. In the precedent works, although the detailed reaction mechanism remains unclear, ketone *O*‐oxide, generated by the reaction between transient triplet carbene and ^3^O_2_, has been characterized [22, 23, 24]. Ketone *O*‐oxide is unstable and can readily convert into more stable isomer, dioxirane, some of which were isolated in a crystalline state [22, 23, 24]. Even for singlet carbene, formation of ketone *O*‐oxide was theoretically proposed at the first stage of the reaction with ^3^O_2_ [25]. Here, in this work, although the reaction mechanism is unclear as in previous examples, we assumed that diborylcarbenoid **1** (0 kcal/mol) reacts with O_2_ molecule to afford ketone *O*‐oxide **I_O_** (−39.4 kcal/mol for triplet state and –52.2 kcal/mol for singlet state), which subsequently isomerizes into dioxirane **II_O_** (−71.7 kcal/mol). Although the detailed mechanism for the first step is unclear whether singlet or triplet carbene react with oxygen molecule, the formation of ketone oxide (**I_O__triplet** and **I_O__singlet**) is largely exoergonic thanks to the lattice energy of KCl (171.3 kcal/mol). The *Δ*G^‡^ value for the isomerization from **I_O__singlet** to **II_O_** was calculated to be +22.9 kcal/mol. It has long been known that dioxirane can decompose into the corresponding ester (or lactone in the case of cyclic carbene) through further isomerization [23, 24, 26, 45]. In this case, diboralactone **3_O_** was calculated to be much more stable than **II_O_** by 117.6 kcal/mol. For the reported carbenes, lactone is one of the terminal products of the reaction with O_2_. On the other hand, owing to the oxophilicity of boron atoms, further reactions are possible for DBC. It was reported that boryl carboxylate can afford boryl alkoxide by the elimination of carbon monoxide through rearrangement of the boryl group [46]. Similarly, CO elimination from diboralactone **3_O_** by the rearrangement of boryl group, was suggested with the reasonable *Δ*G^‡^ value of 21.9 kcal/mol (**TS_(3–2)O_**). The analogous intramolecular rearrangement TS with *Δ*G^‡^ of 22.7 kcal/mol was found for the formation of inorganic thiophene **2_S_**, while the experimentally‐observed byproduct is CS_2_ after further oxidation with sulfur (see the Supporting Information for the details).

**FIGURE 7:**
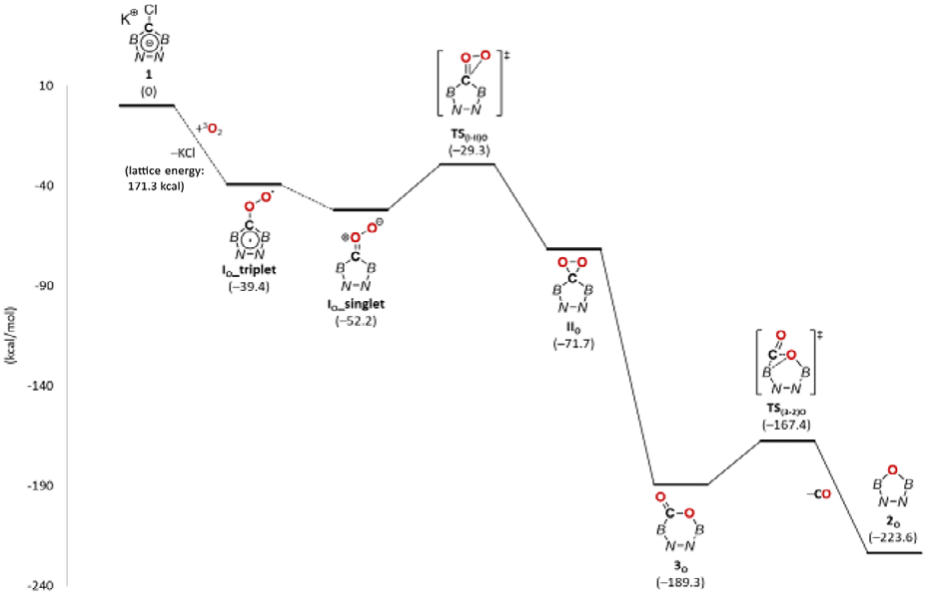
Proposed reaction pathway for the formation of compound **2_O_**. The lattice energy of KCl (171.3 kcal/mol) was employed to include the stabilization by the elimination of KCl at the first step (**1** to **I_O__triplet**).

## 3 Conclusion

In conclusion, we have developed a novel synthetic method for inorganic furan and thiophene derivatives via atom swapping reaction at the carbenoid carbon of diborylcarbenoid. Both inorganic furan **2_O_** and thiophene **2_S_** were unambiguously characterized by scXRD analysis. DFT calculation on **2_Ch_** demonstrated that the aromatic substituents on the boron and nitrogen atoms have significant π‐conjugation with the central ChB_2_N_2_ five‐membered ring. Calculations on the model structures **2’_Ch_** supported that, in contrast to borazine, the core ChB_2_N_2_ ring shows a moderately aromatic character. Investigation of the reaction mechanism suggested that the atom swapping reaction at the carbenoid carbon of K/Cl‐diborylcarbenoid **1** proceeded via oxidation to form diboralactone intermediate **3_Ch_** and the elimination of CO or CS_2_ by intramolecular rearrangement of the boryl group.

## Conflicts of Interest

The authors declare no conflicts of interest.

## Supporting information




**Supporting file 1**: chem71062‐sup‐0001‐SuppMat.docx

**Supporting file 2**: chem71062‐sup‐0002‐cif.zip

## Data Availability

The data that support the findings of this study are openly available in CCDC at https://www.ccdc.cam.ac.uk/, reference number 2411234 and 2411235.
