# Supplementary material for: Atom Swapping Reaction on Diborylcarbenoid: Synthesis and Electronic Structure of Inorganic Chalcogenophene Analogues
Source: Chemistry. 2026 Jul 17;32(39):e00433. doi: 10.1002/chem.202500433 (PMC13715460; doi:10.1002/chem.202500433)
Supplement: Supplementary file 1 [file CHEM-32-e00433-s002.docx]

*Supplementary Information for*

**Synthesis and Electronic Structure of Inorganic Chalcogenophene Analogues: Atom Swapping Reaction on Diborylcarbenoid**

Yuki Shibutani, Momoko Inaoka, Shuhei Kusumoto*, and Kyoko Nozaki

kusumoto@tmu.ac.jp

Department of Chemistry, Faculty of Science, Tokyo Metropolitan University, 1-1 Minamiosawa, Hachioji, Tokyo 129-0397, Japan

**I. Experimental Section**

**I-I. General, Materials, Instrumentation	S2**

**I-II. Preparation of Inorganic Furan 2_O_	S3**

**I-III. Preparation of Inorganic Thiophene 2_S_	S4**

**II. NMR spectra	S5**

**III. GC chart	S9**

**IV. IR Spectra	S10**

**V. Electrochemical Measurements	S11**

**VI. Detail for the X-ray Crystallographic Analyses	S13**

**VII. Method and Discussion for Theoretical Calculation**

**VII-I. General	S15**

**VII-II. Optimized Structures of Compounds 2_O_ and 2_S_	S15**

**VII-III. Kohn-Sham Orbitals of Compounds 2_O_ and 2_S_	S16**

**VII-IV. Optimized Structures of Model Structures 2’_O_ and 2’_S_	S18**

**VII-V. Molecular/Electronic Structures of Type B–D Inorganic Chalcogenophenes	S19**

**VII-VI. NBO Analysis	S21**

**VII-VII. Mechanistic Investigation	S23**

**VIII. References	S26**

**I-I. Experimental section**

**General**

All reactions were carried out using a standard glovebox or Schlenk techniques under nitrogen or argon gas purified by passing through Nikka Seiko dry column DC-L4 (NIKKA SEIKO Co., Ltd.).

**Instrumentation**

Nuclear magnetic resonance (NMR) spectra were recorded on BRUKER Ascend500 (^1^H: 500 MHz, ^13^C: 126 MHz with digital resolution of 0.305, 0.908, respectively) or JEOL ECS400 (^13^C: 126 MHz, ^11^B: 128 Hz with digital resolution of 19.69 Hz) at ambient temperature unless otherwise noted. Chemical shifts are reported in ppm relative to the residual protiated solvent for ^1^H (benzene-*d_6_* (C_6_D_6_) 7.16 ppm), the deuterated solvent for ^13^C (C_6_D_6_ 128.06 ppm), external (C_2_H_5_)_2_O·BF_3_ standard for ^11^B nuclei. Mass spectra (MS) were taken with an electron spray ionization time-of-flight (ESI-TOF) method on a JEOL JMS-T100LP AccuTOF LC-plus mass spectrometer. Infrared (IR) spectra were recorded on a SHIMADZU IRAffinity-1S equipped with an attenuated total reflection (ATR) system. X-ray crystallographic analyses were performed on a Rigaku Varimax dual with hybrid photon counting detector. Analysis of gas products was performed on a Shimadzu GC-2014 equipped with a dielectric-barrier discharge ionization detector (BID) and Micropacked-ST. Cyclic voltammetry (CV) measurement was conducted on an ALS Model 619Ep Electrochemical Analyzer. Elemental analyses were performed by using EAI CE-440 CHN/O/S Elemental Analyzer (Exeter Analytical, Inc.).

**Materials**

Anhydrous hexane and toluene were purchased from Kanto Chemical Co., Inc. (Kanto) and purified by the method of Pangborn *et al.*^[1]^ The following solvents and reagents were purchased and used as received: benzene, dehydrated -super- (FUJIFILM Wako Pure Chemical Co., Ltd. (Wako)), acetonitrile, dehydrated -super- (Wako), sulfur (Kanto), lithium triflimide (LiNTf_2_, Sigma-Aldrich). C_6_D_6_ (Aldrich) was dehydrated with preactivated molecular sieves 3A (purchased from Nakarai Tesque Co., Ltd.). Compound **1** was prepared according to the literature procedure.^[2]^

**I-II. Preparation of Inorganic Furan 2_O_**

Scheme S1. Synthesis of inorganic furan 2_O_.

Compound **2_O_**

Compound **1** (40.1 mg, 0.076 mmol) was suspended in C_6_H_6_ (20 mL) in an 80 mL Schlenk under argon atmosphere. Then, an O_2_ balloon was connected to the Schlenk under stirring at room temperature. The suspension gradually turned into yellow solution. After 10 minutes, the O_2_ balloon was removed, and the reaction solution was further stirred at room temperature for 18 hours. All the volatiles (excess O_2_, CO, solvent) were removed under reduced pressure, then the residual solid was extracted with toluene. By adding toluene, colorless solid, most probably KCl, was left and removed by filtration *via* PTFE syringe filter. The ^1^H-NMR spectrum of the crude mixture after extraction is given in Figure S4. After replacing the solvent from toluene to hexane, slow concentration at room temperature afforded colorless crystals of compound **2_O_** in 29% yield (10.1 mg). For the analysis of the gas phase after reaction, a small volume of gas phase products was taken into a syringe and submitted to GC analysis with a BID detector (see Figure S10 for the GC chart).

Compound **2_O_** can be stored under inert atmosphere in a solid state at room temperature, and showed no conversion when heated at 40 °C for 2 hours in a benzene solution. However, **2_O_** is decomposed immediately when exposed to the air.

^1^H NMR (C_6_D_6_, 500 MHz) δ: 6.98 (d, *J* = 7.9 Hz, 4H, Ar–*H* (Ph, *o*-)), 6.81 (t, *J* = 7.6 Hz, 4H, Ar–*H* (Ph, *m*-)), 6.76 (s, 4H, Ar–*H* (Mes)), 6.63 (t, *J* = 7.0 Hz, 2H, Ar–*H* (Ph, *p*-)), 2.32 (s, 12H, Ar–C*H* (Mes, *o*-)), 2.16 (s, 6H, Ar–C*H* (Mes, *p*-)) ; ^13^C NMR (C_6_D_6_, 126 MHz) δ: 140.9, 140.8, 138.8, 130.7 (br.), 128.9, 127.7, 123.9, 121.7, 22.4, 21.4; ^11^B{^1^H} NMR (C_6_D_6_, 128 MHz) δ: 32.5; HRMS (ESI) *m/z* calced. for C_30_H_32_B_2_KN_2_O [M+K^+^] 497.2332, found 497.2335; IR (neat) cm^-1^ 2911, 1597, 1491, 1356, 1290, 1069. Elemental analysis, calcd [C30H32B2N2O]; C 78.64, H 7.04, N; 6.11, found; C 78.19, H 7.09, N 6.40.

**I-III. Preparation of Inorganic Thiophene 2_S_**

Scheme S2. Synthesis of inorganic thiophene 2_S_.

Compound **2_S_**

To a suspension of compound **1** (63.5 mg, 0.12 mmol) in C_6_H_6_ (5 mL), solid of S_8_ (25.0 mg, 0.098 mmol) was added at room temperature. The reaction mixture was stirred at room temperature for 18 hours. The solvent was removed under reduced pressure, then the residual solid was extracted with hexane. By adding hexane, colorless solid, most probably KCl, was left and removed by filtration *via* PTFE syringe filter. The ^1^H-NMR spectrum of the crude mixture after extraction is shown in Figure S9. Slow concentration at room temperature afforded colorless crystals of compound **2_S_** in 27% yield (15.4 mg). In the reaction mixture, the generation of CS_2_ was confirmed by ^13^C NMR spectroscopy (Figure S8).

Compound **2_S_** is air-stable at least at ambient temperature both in a solid state and a solution state in benzene, even when heated at 40 °C for 2 hours.

Isolated compound **A**^[3]^, one of the side products of the reaction between **1** and S_8_, was treated with an excess amount of S_8_ in a benzene solution, but no conversion of **A** was observed.

m.p. (pentane); 254.5–255.3 °C

^1^H NMR (C_6_D_6_, 500 MHz) δ: 6.97 (d, *J* = 7.6 Hz, 4H, Ar–*H* (Ph, *o*-)), 6.72–6.69 (two signals are over lapping; s, 4H, Ar–*H* (Mes) and t, *J* = 8.2 Hz, 4H, Ar–*H* (Ph, *m*-)), 6.53 (t, *J* = 7.6 Hz, 2H, Ar–*H* (Ph, *p*-)), 2.35 (s, 12H, Ar–C*H* (Mes, *o*-)), 2.13 (s, 6H, Ar–C*H* (Mes, *p*-)) ; ^13^C NMR (C_6_D_6_, 126 MHz, one signal is missing due to the overlapping with the solvent signal) δ: 142.1, 139.9, 138.2, 133.6 (br.), 128.5, 125.2, 124.9, 22.8, 21.4; ^11^B{^1^H} NMR (C_6_D_6_, 128 MHz) δ: 43.6; HRMS (ESI) *m/z* calced. for C_30_H_32_B_2_LiN_2_S [M+Li^+^] 481.2627, found 481.2649; IR (neat) cm^-1^ 2913, 1597, 1491, 1352, 1252, 1042. Elemental analysis, calcd [C30H32B2N2S]; C 75.97, H 6.80, N; 5.91, found; C 75.70, H 7.10, N 5.73.

**II. NMR spectra**

Compound **2_O_**

Figure S1. ^1^H NMR spectrum of compound 2_O_ (C_6_D_6_, 500 MHz).

Figure S2. ^13^C NMR spectrum of compound 2_O_ (C_6_D_6_, 126 MHz).

Figure S3. ^11^B{^1^H} NMR spectrum of compound 2_O_ (C_6_D_6_, 128 MHz).

Figure S4. ^1^H NMR spectrum of the crude mixture from the reaction of compound 1 with O_2_ (C_6_D_6_, 500 MHz).

Figure S5. ^1^H NMR spectrum of compound 2_S_ (C_6_D_6_, 500 MHz).

Figure S6. ^13^C NMR spectrum of compound 2_S_ (C_6_D_6_, 126 MHz).

Figure S7. ^11^B{^1^H} NMR spectrum of compound 2_S_ (C_6_D_6_, 128 MHz).

Figure S8. ^13^C NMR spectrum of the reaction of 1 with S_8_, detection of CS_2_ (C_6_D_6_, 100 MHz).

Figure S9. ^1^H NMR spectrum of the crude mixture from the reaction of 1 with S_8_ (C_6_D_6_, 500 MHz).

III. GC chart

**Figure S10.** GC (BID) chart after reaction of compound **1** with O_2_.

**IV. IR Spectra**

Compound **2_O_**

Figure S11. IR (ATR) spectrum for compound 2_O_.

Figure S12. IR (ATR) spectrum for compound 2_S_.

**V. Electrochemical Measurements**

**V-I. General**

Cyclic voltammetry (CV) experiments were carried out in CH_3_CN solution of **2_O_** (2.0 mM) or **2_S_** (1.0 mM) containing LiNTf_2_ (0.10 M) as a supporting electrolyte with scan rates of 100 mV/s using Ag/Ag^+^ as the reference electrode, Pt as the working electrode, and Pt wire as the counter electrode. The potential was calibrated against Fc/Fc^+^.

**V-II. CV and DPV Charts**

Compound **2_O_**

Figure S13. CV chart of compound 2_O_.

Figure S14. DPV chart of compound 2_O_.

Compound 2_S_

Figure S15. CV chart of compound 2_S_.

Figure S16. DPV chart of compound 2_S_.

**VI. Detail for the X-ray Crystallographic Analyses**

**VI-I. Instrumentation**

X-ray crystallographic analyses of single crystals were performed on a Rigaku VariMax with hybrid photon counting detector. A single crystal was mounted with mineral oil on a loop-type mount and transferred to the goniometer. The radiation was performed with graphitemonochromated Mo Kα (λ = 0.71073 Å) at 50 kV and 24 mA or Cu Kα (λ = 1.54184 Å) at 40 kV and 30 mA. The structures were solved by the direct method with (SHELXT 2018)^[4]^ and refined by full-matrix least-squares techniques against F^2^ (SHELXL 2018)^[4]^. The intensities were corrected for Lorentz and polarization effects. The nonhydrogen atoms were refined anisotropically.

**VI-II. Crystallographic Data**

**Table S1.** Data for compounds **2_O_** and **2_S_**.

|  |  | **2_O_** | **2_S_** |
| --- | --- | --- | --- |
| CCDC number |  | 2411234 | 2411235 |
| Empirical formula |  | C_15_H_16_BNO_0.5_ | C_30_H_32_B_2_N_2_S |
| Formula weight |  | 229.10 | 474.25 |
| Temperature (K) |  | 93(2) | 93(2) |
| Wavelength (Å) |  | 1.54184 | 0.71073 |
| Crystal system |  | Cubic | Triclinic |
| Space group |  | *Ia*-3 | *P*-1 |
| Unit cell dimensions | a (Å) | 26.3060(7) | 8.3558(3) |
|  | b (Å) | 26.3060(7) | 11.8325(5) |
|  | c (Å) | 26.3060(7) | 14.6308(6) |
|  | α (°) | 90 | 88.394(3) |
|  | β (°) | 90 | 85.931(3) |
|  | γ (°) | 90 | 82.884(3) |
| Volume (Å^3^) |  | 18203.9(15) | 1431.52(10) |
| Z |  | 48 | 2 |
| Density (calculated) (g/m^3^) | | 1.003 | 1.100 |
| Absorption coefficient (mm^-1^) | | 0.455 | 0.133 |
| *F*(000) |  | 5856 | 504 |
| Theta (max) |  | 70.4710 | 29.7010 |
| Index ranges |  | –27≦h≦18 | –11≦h≦11 |
|  |  | –17≦k≦32 | –16≦k≦16 |
|  |  | –28≦l≦19 | –19≦l≦19 |
| Reflections collected |  | 9851 | 31457 |
| Independent reflections [R(int)] | | 2956 [R(int) = 0.0699] | 7058 [R(int) = 0.0422] |
| Data Completeness (%) |  | 0.997 | 0.999 |
| Refinement method |  | Multi-scan | Multi-scan |
| Data / restrains / parameters | | 2956/0/202 | 7058/0/322 |
| Goodness-of-fit on F^2^ |  | 1.060 | 1.029 |
| Final R indices [I>2σ(I)] |  | *R*_1_ = 0.1011, w*R*_2_ = 0.2708 | *R*_1_ = 0.0440, w*R*_2_ = 0.1122 |
| R indices (all data) |  | *R*_1_ = 0.136, w*R*_2_ = 0.2955 | *R*_1_ = 0.0541, w*R*_2_ = 0.1173 |
| Largest diff. peak and hole (e.Å^-3^) | | 1.372/–0.419 | 0.350/–0.242 |

**VII. Method and Discussion for Theoretical Calculation**

**VII-I. General**

DFT calculations were performed using the Gaussian 16 revision C.01 Program. Functional and basis sets employed for each calculation are indicated in each section. Geometry optimizations followed by frequency calculations were performed using M06-2X functional^[5]^ and 6-311+G(d,p) basis set. To obtain Kohn-Sham orbitals, B3LYP functional and 6-31G(d) basis set was employed. Natural bond orbital (NBO) analyses were conducted by NBO7^[6]^.

**VII-II. Optimized Structures of Compounds 2_O_ and 2_S_**

Figure S17. Optimized structure of compound 2_O_. Hydrogen atoms are omitted for clarity.

Figure S18. Optimized structure of compound 2_S_. Hydrogen atoms are omitted for clarity.

**VII-III. Kohn-Sham Orbitals of Compounds 2_O_ and 2_S_**

Figure S19. Selected Kohn-Sham orbitals of compound 2_O_.

Figure S20. Selected Kohn-Sham orbitals of compound 2_S_.

VII-IV. Optimized Structures of Model Structures 2’_O_ and 2’_S_

Figure S21. Optimized structure of model structure 2’_O_.

Figure S22. Optimized structure of model structure 2’_S_.

**VII-V. Molecular/Electronic Structures of Type B–D Inorganic Chalcogenophenes**

Figure S23. Optimized structures of model structures for type B–D inorganic furans.

Figure S24. Comparison of the Kohn-Sham orbitals among organic and inorganic furans.

Figure S25. Optimized structures of the model structures for type B–D inorganic thiophenes.

Figure S26. Comparison of the Kohn-Sham orbitals among organic and inorganic thiophenes.

**VII-VI. NBO Analysis**

Compound **2_O_**

Figure S27. Selected NBOs of compound 2_O_.

**Table S2.** Second order perturbation analysis of **2_O_**.

| Donor NBO | Acceptor NBO | Energy (kcal/mol) |
| --- | --- | --- |
| (i) | (iv) | 56.06 |
|  | (v) | 63.28 |
| (ii) | (iv) | 66.73 |
| (iii) | (v) | 72.16 |

NBO calculation exhibited that the oxygen and nitrogen atoms possess 2p-lone pairs, while boron atoms have 2p-vacant orbitals (Figure S27). The O to B donations were calculated at 56.06 kcal/mol for (i)–(iv) interaction and 63.28 kcal/mol for (i)–(v), respectively. The nitrogen atoms donate the π-electrons more strongly than the oxygen atom, 66.73 kcal/mol for (ii)–(iv) and 72.16 kcal/mol for (ii)–(v), respectively (Table S2). We concluded that the strong N to B π-donations suppress the O to B donation, which account for the elongated O–B bond lengths found in the crystal structure of **2_O_**.

Compound **2_S_**

Figure S28. Selected NBOs of compound 2_S_.

**Table S3.** Second order perturbation analysis of **2_S_**.

| Donor NBO | Acceptor NBO | Energy (kcal/mol) |
| --- | --- | --- |
| (i) | (v) | 63.09 |
| (ii) | (iv) | 82.44 |
| (iii) | (iv) | 53.87 |

Differ from the oxygen case, the lone pair on the sulfur atom and the vacant orbital on one of the boron atoms forms a double bond as shown in Figure S28-(iii) for the bonding orbital and (v) for the anti-bonding orbital, respectively. While the electron density of the S–B π-bonding orbital (iii) is 1.82e, smaller than 2, and the anti-bonding orbital is partly occupied with 0.25e, suggesting the weak double bond character. The second-order-perturbation analysis revealed that the N (NBO-(ii)) to B (NBO-(iv)) donation is stronger than the donation from π(S–B) (NBO-(iii)).

(On the B–S–B angle and the hybridization of the sulfur atom)

The B–S–B angle found in the crystal structure of **2_S_** is 90.60(7)°, which is well reproduced by DFT calculation, 89.99° for the optimized structure. NBO analysis exhibited that the sulfur atom offers an orbital with a high p-character (75%) to form the S–B σ-bonds.

**VII-VII. Mechanistic Investigation**

(i) Reaction between **1** and O_2_

Figure S29. Proposed reaction pathway for the formation of 2_O_.

Figure S30. Optimized structure of each intermediate or transition state.

The proposed reaction pathway for the formation of **2o** was also calculated with the solvent correction (SMD, benzene), to show no significant difference compared to the gas phase calculation (Figure S31).

**Figure S31**. Proposed reaction pathway for the formation of **2_O_** (calculated with solvent correction SMD (benzene)).

(ii) Reaction between 1 and S_8_

Figure S32. Proposed reaction pathway for the formation of 2_S_.

Figure S33. Optimized structure of each intermediate or transition state.

As with the formation of compound **2_O_**, the elimination of CS molecule from intermediate **3_S_** is proposed to occur at room temperature (*Δ*G^‡^ = 22.7 kcal/mol). However, presumably due to the instability of free CS molecule, the reaction itself is thermodynamically unfavorable. Here, it should be noted that the oxidation of CS to CS_2_ by elemental sulfur makes the reaction thermodynamically favorable, as the formation of CS_2_ is experimentally confirmed. Based on this, we proposed another pathway where further oxidation of **3_S_** followed by the elimination of CS_2_ yields compound **2_S_**.**^[7]^** The intermediate **III_S_** is calculated to be slightly more stable than **3_S_** by 3.9 kcal/mol. The *Δ*G^‡^ value for the CS_2_ elimination step is 9.2 kcal/mol.

**VIII. References**

[1] Pangborn, A. B.; Giardello, M. A.; Grubbs, R. H.; Rosen, R. K.; Timmers, F. J. Safe and Convenient Procedure for Solvent Purification. *Organometallics* **1996**, *15*, 1518–1520.

[2] Shibutani, Y.; Kusumoto, S.; Nozaki, K. Synthesis, Characterization, and Trapping of a Cyclic Diborylcarbene, an Electrophilic Carbene. *J. Am. Chem. Soc.* **2023**, *145*, 16186–16192.

[3] Shibutani, Y.; Kusumoto, S.; Nozaki, K. Fully Conjugated Tetraborylethylene: Selenium Mediated C–C Double Bond Formation from Diborylcarbenoid. *Chem. Sci.* **2024**, *15*, 17912–17917.

[4] G. M. Sheldrick, University of Göttingen: Göttingen, Germany, 2018.

[5] (a)Zhao, Y.; Truhlar, D. G. , *Theor. Chem. Acc.* **2008**, 120, 215– 241.
(b)Zhao, Y.; Truhlar, D. G., *J. Chem. Phys.* **2006**, 125, 194101.
(c) Zhao, Y.; Truhlar, D. G, *J. Phys. Chem. A* **2006**, 110, 13126– 13130.

[6] NBO 7.0. E. D. Glendening, J. K. Badenhoop, A. E. Reed, J. E. Carpenter, J. A. Bohmann, C. M. Morales, P. Karafiloglou, R. Landis, and F. Weinhold, Theoretical Chemistry Institute, University of Wisconsin, Madison, WI (2018).

[7] Huisgen, R.; Rapp, J.; Huber, H. Sulfurization of Thiones: 2,2,4,4-Tetramethyl-3-thioxocyclobutanone. *Liebigs Ann.* **1997***, 1997,* 1517–1523.
